# Influence of age on histologic outcome of cervical intraepithelial neoplasia during observational management: results from large cohort, systematic review, meta-analysis

**DOI:** 10.1038/s41598-018-24882-2

**Published:** 2018-04-23

**Authors:** Christine Bekos, Richard Schwameis, Georg Heinze, Marina Gärner, Christoph Grimm, Elmar Joura, Reinhard Horvat, Stephan Polterauer, Mariella Polterauer

**Affiliations:** 1 Medical University of Vienna, Comprehensive Cancer Center, Department of Obstetrics and Gynecology, Division of General Gynecology and Gynecologic Oncology, Viennna, Austria; 2 Center for Medical Statistics, Informatics and Intelligent Systems, Medical University of Vienna, Viennna, Austria; 3 Medical University of Vienna, Department of Pathology, Viennna, Austria; 4 Karl Landsteiner Institute for General Gynecology and Experimental Gynecologic Oncology, Viennna, Austria

## Abstract

Aim of this study was to investigate the histologic outcome of cervical intraepithelial neoplasia (CIN) during observational management. Consecutive women with histologically verified CIN and observational management were included. Histologic findings of initial and follow-up visits were collected and persistence, progression and regression rates at end of observational period were assessed. Uni- and multivariate analyses were performed. A systematic review of the literature and meta-analysis was performed. In 783 women CIN I, II, and III was diagnosed by colposcopically guided biopsy in 42.5%, 26.6% and 30.9%, respectively. Younger patients had higher rates of regression (p < 0.001) and complete remission (< 0.001) and lower rates of progression (p = 0.003). Among women aged < 25, 25 < 30, 30 < 35, 35 < 40 years, and > 40 years, regression rates were 44.7%, 33.7%, 30.9%, 27.3%, and 24.9%, respectively. Pooled analysis of published data showed similar results. Multivariable analysis showed that with each five years of age, the odds for regression reduced by 21% (p < 0.001) independently of CIN grade (p < 0.001), and presence of HPV high-risk infection (p < 0.001). Patient’s age has a considerable influence on the natural history of CIN – independent of CIN grade and HPV high-risk infection. Observational management should be considered for selected young patients with CIN.

## Introduction

The estimated annual incidence of cervical intraepithelial neoplasia (CIN) among women who undergo cervical cancer screening is 4% for CIN I and 5% for CIN II, III^1^. The societal importance is accentuated by the peak of annual incidence in young women aged 20 to 24 years for CIN I (5.1 per 1,000) whereas rates of CIN II (3.8 per 1,000) and CIN III (4.1 per 1,000) peak in the 25 to 29 age group^2^.

In general, CIN can either resolve spontaneously or persist or progress when not treated immediately. CIN II, III are associated with a risk of developing cervical cancer, and are typically treated with conisation. However, there is some chance that these lesions will regress, and observation can be chosen for selected patients. This is particularly an option for women who plan future childbearing, since excisional procedures had been related to an increased risk for adverse pregnancy outcomes^3^.

The strongest factor influencing the natural history of CIN is the presence of high-risk human papillomavirus (HPV) infection. In particular HPV 16 and 18 increase the risk for persistent disease^4^. Further, smoking^5^, multiparity and long-term use of oral contraceptives can double or triple the risk for progression to high-grade lesions or cervical cancer in HPV infected women^4–6^. Several studies have suggested that patient’s age is an independent factor influencing regression and progression rates of CIN^7,8^. Younger women generally seem to have higher rates of spontaneous regression and remission^9,10^. Therefore, recent guidelines adapted these findings and recommend observational management as an option in young women with CIN^11,12^. However, most of these studies reported mainly cytological changes and had no serial histologic outcome data available.

The aim of this study was to evaluate the natural history of CIN lesions and to assess histological progression and regression rates in different age groups using colposcopically guided biopsy results. In addition, a systematic review of the literature and meta-analysis of published data was performed^13^.

## Results

In total 852 patients were identified to have CIN I-III on colposcopically guided biopsy and of those 788 met the inclusion criteria. Five of these patients were excluded due to missing variables and a total of 783 were eligible for final analysis.

Table 1 shows patient’s characteristics of the study population. The mean (SD) age at diagnosis was 33.41 (9.53) years. A total of 144 (18.4%), 176 (22.5%), 153 (19.5%), 141 (18.0%) and 169 (21.6%) women <25, 25 < 30, and >30 years respectively were included. At time of CIN diagnosis 42.5% presented with CIN I, 26.6% with CIN II, and 30.9% with CIN III, respectively. Information on HPV status was available for 558 patients and was present in 86.6% of patients with data available. Mean (SD) follow-up time during observational management was 28.8 (30.7) months. Mean (SD) time to regression and complete remission of CIN was 8.3 (5.5) and 9.5 (7.4) months, respectively.

**Table 1:** Patients’ characteristics (N = 783).

Parameter	N (%)
**Histology at first visit**	
CIN I	333 (42.5)
CIN II	208 (26.6)
CIN III	242 (30.9)
**Age**	
< 25	141 (18.0)
25 < 30	178 (22.7)
30 < 35	152 (19.4)
35 < 40	139 (17.8)
> 40	173 (22.1)
**HPV**	
High-risk positive	483 (61.7)
High-risk negative	75 (9.6)
N/A	225 (28.7)
**Smoking Status**	
Smoker	256 (32.7)
Non- or Exsmoker	193 (24.6)
N/A	334 (42.7)
**Contraception**	
Condom	76 (9.7)
Other barrier method	40 (5.1)
Oral contraceptive	152 (19.4)
IUD	42 (5.4)
Other non-barrier	8 (1.0)
No contraception	169 (21.6)
N/A	296 (37.8)
**Parity**	
Nullipara	223 (28.5)
Primipara	141 (18.0)
Multipara	201 (25.7)
N/A	218 (27.8)
**Sexual partners (N)**	
0–5	103 (13.2)
6–10	60 (7.7)
> 10	74 (9.5)
N/A	546 (69.7)
**Socioeconomic status**	
Basic education	122 (15.6)
High school education	89 (11.4)
College education	46 (5.9)
N/A	526 (67.2)
**Treatment**	
Conservative	442 (56.4)
Immediate	341 (43.6)
**Immunosuppression**	
Immunocompetent	760 (97.1)
Immunosuppressive therapy	16 (2.0)
Immunosuppressive diseases	7 (0.9)

CIN, cervical intraepithelial neoplasia; HPV, human papilloma virus; N/A, not available; IUD, intrauterine device.

In Table 2 the rates of histological persistence, progression, regression, and complete remission of CIN diagnosed by colposcopically guided biopsy at the end of follow-up are provided.

**Table 2:** Histologic outcome of the complete study cohort (N = 783).

Age (years)	N	Status at last follow-up			
		Regression (%)	Persistence (%)	Progression (%)	Complete Remission (%)
<25	141	63 (44.68)	63 (44.68)	15 (10.64)	56 (39.72)
≥25 < 30	178	60 (33.71)	94 (52.81)	24 (13.48)	52 (29.21)
≥30 < 35	152	47 (30.92)	79 (51.97)	26 (17.11)	39 (25.66)
≥35 < 40	139	38 (27.34)	86 (61.87)	15 (10.79)	31 (22.30)
≥40	173	43 (24.85)	87 (50.29)	43 (24.86)	40 (23.12)
P-value for trend		<0.001	0.174	0.003	<0.001

N, number.

In Table 3 the rates of histological persistence, progression, regression, and complete remission of CIN are shown for patients managed conservatively for at least 3 months (n = 442).

**Table 3:** Histologic outcome after observational management of the study cohort (N = 442).

Age (years)	N	Status at last follow-up			
		Regression (%)	Persistence (%)	Progression (%)	Complete Remission (%)
<25	111	64 (57.7)	33 (29.7)	14 (12.6)	56 (50.5)
≥25 < 30	107	56 (52.3)	37 (34.6)	14 (13.1)	50 (46.7)
≥30 < 35	85	41 (48.2)	34 (40.0)	10 (11.8)	36 (42.4)
≥35 < 40	61	33 (54.1)	24 (39.3)	4 (6.6)	31 (50.8)
≥40	78	39 (50.0)	22 (28.2)	17 (21.8)	38 (48.7)
P-value for trend		0.351	0.832	0.283	0.918

N, number.

In Table 4 histologic outcome rates after observational management categorized by duration of observational management are presented.

**Table 4:** Histologic outcome after observational management of the study cohort (N = 442) categorized by duration of observational management.

Follow-up time (months)	N	Status at last follow-up			
		Regression (%)	Persistence (%)	Progression (%)	Complete Remission (%)
≥3 < 9	213	100 (46.9)	81 (38.0)	32 (15.0)	91 (42.7)
≥9 < 15	105	59 (56.2)	31 (29.5)	15 (14.3)	54 (51.5)
≥15 < 21	69	39 (56.5)	21 (30.4)	9 (13.0)	33 (47.8)
≥21 < 27	40	24 (60.0)	13 (32.5)	3 (7.5)	23 (57.5)
P-value for trend		0.212	0.408	0.647	0.243

N, number.

**Figure 1:**
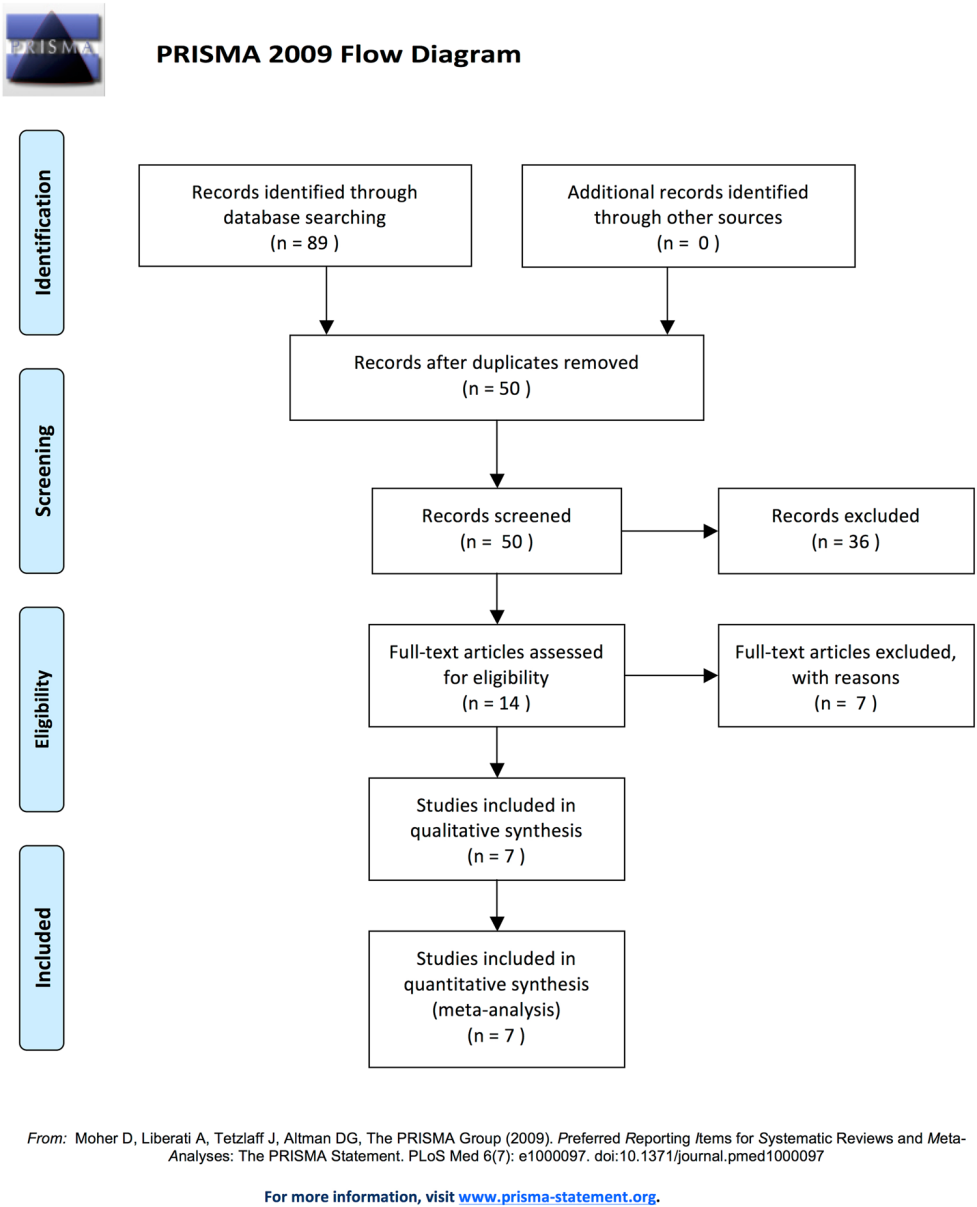
PRISMA (Preferred Reporting Items for Systematic Reviews and Meta-Analyses) flow diagram for study selection^20^.

In Table 5 overall regression, persistence, and progression rates revealed by pooled analysis using a random effects model are shown. Data from cited studies that had information for regression, progression, and regression were included into the pooled analysis for each age group. Not all studies had information for all age groups available. The data of our cohort were included into all analyses. In total 7 articles (Table S1 in supplemental material) were found matching the search criteria^12^. Before excluding studies lacking histological diagnosis and providing only cytological results 14 studies were identified. PRISMA (Preferred Reporting Items for Systematic Reviews and Meta-Analyses) flow diagram for study selection is shown in Fig. 1. Only data from studies where histological baseline and outcome information was available were summarized together with the present study within the pooled analysis. Seven articles meeting the inclusion criteria were identified and included into the pooled analysis. Regression rates among the studies included varied between 38.9% and 76.2%. For a summary of all included studies please find Table S1 (supplemental material) attached. Risk of bias assessment revealed some evidence for attrition bias in four out of seven studies (see Supplemental material, Table S5–S12). However, meta-regression could not provide evidence for an impact of median follow-up on regression rates (odds ratio per month, 1.02, 95%CI 0.99–1.05, p = 0.23). Publication bias (evidenced by smaller studies reporting higher regression or persistence or lower progression rates) was not present (see data shown in Supplemental Material).

**Table 5:** Pooled analysis of studies reporting age- dependent outcome rates of CIN after observational management.

Age	Pooled analysis											
	Regression				Persistence				Progression			
	N*	Rate (%)	95% CI	I^2^%	N*	Rate (%)	95% CI	I^2^%	N*	Rate (%)	95% CI	I^2^%
<25	754^10,14–19^	58.4	53.1–63.5	67.7	755^15,16,18,19^	31.3	22.4–41.9	77.3	561^10,19^	13.9	11.3–17.0	0
<30	938^10,14,15,17–19^	52.9	44.3–61.3	82.6	832^15,16,18,19^	32.1	23.5–42.1	76.2	668^10,19^	13.8	11.4–16.6	0
<35	1058^10,14,15,17–19^	51.9	43.3–60.5	84.3	999^15,16,18,19^	34.5	31.6–37.5	0	753^10,19^	13.5	11.3–16.2	0
≥35	172^17^	46.2	32.7–60.3	33.4	197^16^	32.5	26.3–39.4	0	139	15.1	10.0–22.1	0

^*^References of included studies, data from the current report was included into all analyses (Table 3); N, number.

n, number;

In Table 6 overall regression, persistence, and progression rates of patients <25 years stratified by CIN grade revealed from pooled analysis are shown. In this analysis, no heterogeneity was present (*I*^2^ = 0%) and therefore, the random effects model coincides with the fixed effects model.

**Table 6:** Pooled analysis of studies with reported histo- pathological outcome of patients <25 years together with the study cohort (N = 111) stratified by CIN grade.

CIN Grade	Median follow up time (months)	Regression			Persistence			Progression		
		N*	Rate %	95% CI	N	Rate %	95% CI	N	Rate %	95% CI
CIN I	12.46	79	54.7%			31.4%			14.0%	
CIN II^10,18^	N/A	274	62.4%	(56.6–68.0)	179	39.7%	(32.7–47.1)	117	14.5%	(9.2–22.2)
CIN III	10.57	10	17.4%			82.6%			0%	

*References of included studies, data from the current report were included into all analyses (Table 3); N, number; *I*^2^ = 0% for all pooled analysis of CIN II.

In Table 7 overall regression, persistence, and progression rates of patients of all age groups who received conservative treatment for at least three months stratified by CIN grade are shown.

**Table 7:** Histologic outcome after observational management of the study cohort (N = 442) categorized by CIN grade.

CIN Grade	Median follow up time (months)	N	Status at last follow-up			
			Regression (%)	Persistence (%)	Progression (%)	Complete Remission (%)
CIN I	10.0	286	169 (59.1)	77 (26.9)	40 (14.0)	169 (59.1)
CIN II	7.23	95	43 (45.3)	34 (5.8)	18 (18.9)	33 (34.7)
CIN III	7.15	61	21 (34.4)	39 (63.9)	1 (1.6)	14 (23.0)
P-value for trend			0.001	0.000	0.007	0.000

N, number.

Table 8 provides results of multivariable logistic regression analysis including all patients of the study population showing the independent association of age (continuous), CIN grade, and HPV high-risk infection on CIN regression rates. The odds ratio of age (per five years) was 0.79 (95%CI 0.71–0.88), suggesting the conclusion that per each five years increase in age, the odds for regression reduce by 11% independently of CIN grade and HPV high-risk infection status.

**Table 8:** Predictors of CIN regression in 783 patients (regression: N = 251; no regression: 532); results of multivariable logistic regression analysis.

Parameter	Odds Ratio	95% Confidence interval	p-value
**Age (continuous):**			
per 5 years	0.89	0.79–0.99	0.049
**Histology:**			
CIN I	1 (ref.)		
CIN II	0.72	0.43–1.19	0.199
CIN III	0.39	0.21–0.75	0.005
**HPV:**			
high-risk negative	1 (ref.)		
high-risk positive	0.29	0.16–0.55	<0.001
**Treatment:**			
treated within 12 weeks	1 (ref.)		
conservatively managed > 12 weeks	11.9	6.2–23.0	<0.001

CIN, cervical intraepithelial neoplasia; HPV, human papilloma virus; ref., reference level.

Regression rates of 111 patients of the study population younger than 25 who were treated conservatively categorized by several known risk factors are shown in Table 9.

**Table 9:** Regression rates in 111 adolescents (<25 years) (regression: N = 64, no regression: N = 47); treated conservatively (for at least 3 months) broken down by HPV infection, smoking status, type of contraception, parity, number of sexual partners, socio-economic status and immunosuppression.

HPV	Regression N (%)	p-value
High-risk positive	41 (64.1)	0.723
High-risk negative	9 (14.1)	
N/A	14 (21.9)	
**Smoking Status**		0.390
Smoker	20 (37.5)	
Non- or Exsmoker	9 (14.1)	
N/A	35 (54.7)	
**Contraception**		0.288
Condom	10 (23.8)	
Other barrier method	2 (3.1)	
Oral contraceptive	23 (35.9)	
IUD	2 (3.1)	
No contraception	5 (7.8)	
N/A	22 (34.4)	
**Parity**		0.060
Nullipara	34 (53.1)	
Primipara	4 (6.3)	
Multipara	2 (3.1)	
N/A	24 (37.5)	
**Sexual partners (N)**		0.436
0–5	5 (7.8)	
> 5	12 (18.8)	
N/A	47 (73.4)	
**Socioeconomic status**		0.657
Basic education	9 (14.1)	
High school education	8 (12.5)	
College education	1 (1.6)	
N/A	46 (71.9)	
**Immunosuppression**		0.749
Immunocompetent	62 (96.9)	
Immunosuppressive therapy or disease	2 (3.1)	

CIN, cervical intraepithelial neoplasia; HPV, human papilloma virus; N/A, not available; IUD, intrauterine device.

## Discussion

The present study investigated age-dependent regression and progression rates in a large population of 783 patients using histologic data. Using a random effects model results were pooled with previously published reports. We found that regression rates are notably higher in younger women with CIN and showed that the effect is independent of CIN grade or presence of HPV high-risk infection.

Our data support the evidence that regression rates are high in young women, and progression is uncommon. In our cohort we observed notably low rates of disease progression in women younger than 30 and no cases of progression from CIN to invasive disease. The results from the pooled analysis support our findings. Especially for the group of women <30 years the number of patients included into the pooled analysis is high and provides useful information for counseling about the likelihood of natural history of CIN during observational management.

In another pooled analysis evaluation of histologic outcome stratified by CIN grade in adolescent patients (<25 years) was performed. According to the published literature, regression was more likely in patients with low-grade CIN compared to high-grade dysplasia. Still, in this population progression seams to be unlikely.

CIN II, III lesions traditionally had been treated with conisation. However, there is some chance that these lesions will regress, and observation can be chosen for selected patients. This is particularly of interest for young women who plan future childbearing, since excisional procedures can be related to an increased risk for adverse obstetric outcomes^10,15,18,19^. In Austria the age of mothers at time of first birth increased by almost five years within the last 20 years and the median age when women receive their first child is currently 29 years^3^. Although treatment for CIN was shown to not adversely affect fertility, treatment was associated with an increased risk of miscarriage in the second trimester^21^. Further it has been shown that women with CIN have a higher risk for preterm birth. This risk positively correlates with increasing cone depth and is higher for excisional procedures compared to ablation^22^.

Therefore, fertility-sparing and conservative management of CIN and cervical cancer became relevant for more women within the reproductive age and avoiding surgery is often desired^23–25^. In the current management of abnormal cervical cancer screening tests and cancer precursors recommendations for treatment and management of CIN such as published by the American Society of Colposcopy and Cervical Pathology (ASCCP) the treatment advice for women younger than 24 was recently adapted^26^. Other countries recommend observational management for women <30 years^11^. Certainly, several safety issues need to be considered when choosing observational management. First, colposcopy has to be satisfactory to rule out invasion or endocervical disease. Secondly, presence of adenocarcinoma *in situ* has to be ruled out by colposcopically guided biopsy to avoid under-treatment. Observational management for women with CIN III who do not plan future childbearing is generally not recommended. In addition the patient has to fully understand the risk of malignancy if conservative treatment is chosen and has to be compliant to ensure adequate close follow up. Regression rates of 32% to 47% are reported in patients with CIN 3, whereas 12% to 40% can develop invasive cancer if untreated^14,27–31^. In addition, even patients who were treated for CIN can have an increased risk for developing recurrent disease. In a recent review treatment failure was reported in 2 to 9% after LEEP^32^.

Patients are at higher risk for progression if HPV infection with oncogenic HPV subtypes persists for longer than 6 to 12 months^33,34^. In a prospective study, 21% of patients who had high-risk HPV infections over 12 months developed CIN 2 or worse in a follow-up period of 30 months^35^. This is in line with our data showing a mean time to progression in our patients <25 years for CIN I and CIN II of 10.30 and 7.95. In the group of CIN III no progression to invasive cancer was observed.

Recommendations from the American College of Obstetricians and Gynecologists, the American Society for Colposcopy and Cervical Pathology, the American Cancer Society, and the American Society for Clinical Pathology advised screening for at least 20 years following diagnosis for women who have a history of CIN 2, CIN 3, or adenocarcinoma *in situ*, even if they had been treated appropriately or had spontaneous regression of cervical neoplasia^36,37^.

The main limitations of our study include the retrospective design, limited data on HPV status and no information on the specific HPV-type. Moreover, due to retrospective design information about risk factors influencing the natural history of CIN is missing for many patients. In addition, follow up after surgery is missing. However, our study investigated the largest population of age-specific regression and progression rates from a single referral center. We used only histological information from colposcopically guided biopsy to assess changes accurately during the observational period. Most results from previous studies of the natural history of CIN are difficult to interpret because diagnoses depend on cytological rather than histologic testing. Results from the multivariate analysis support the independent effect of age on the natural history of CIN. As typical for meta-analysis including studies with different inclusion criteria and study types, presence of bias cannot be excluded. By applying the Cochrane risk of bias assessment tool, we identified some evidence for attrition bias (different follow-up schemes and lengths of follow-up). In order to take into account the partly considerable heterogeneity among the included studies, in particular for regression and persistence, we performed random effects analyses throughout. Different follow-up lengths and schemes could be a reason for the observed heterogeneity, and our pooled random-effects estimates must be interpreted with caution. They estimate the expected values of regression, persistence and progression for a cohort with average characteristics (e.g., with respect to follow-up) rather than those for the pooled population. We could not find any evidence of publication bias.

In conclusion, our study provides clinically relevant findings on the influence of age on the natural history of CIN during observational management. The rates of regression are notably high in young women with CIN and the risk of progression is low. Observational management should be considered for selected young women who plan future childbearing. Results from the systematic review and meta-analysis of a large data set of new and previously published data can be used for patient selection and counseling.

## Methods

We performed a retrospective analysis of consecutive women who underwent colposcopically guided biopsy at the department of Obstetrics and Gynaecology, Division of Gynaecology and Gynaecological Oncology, General Hospital Vienna, Medical University of Vienna from 2006–2010 after abnormal PAP screening results. Approval for this retrospective study was obtained from the Medical University Vienna Review Board (ECS 1213–2011). Due to the retrospective design of the present study the institution’s IRB granted a waiver of consent and no informed consent was obtained. All patient records were anonymized and de-identified prior to analysis. All patients’ data included in this study are presented in S1 Dataset.

The present study included patients identified via the institution’s electronic documentation system. Data abstracted from medical records included patient demographics, smoking habits, contraception, parity, number of sexual partners, socioeconomic status, biopsy results, treatment of CIN and follow-up biopsy results. The database was searched for women who had received a histologic diagnosis of CIN I–III and were managed conservatively (observation) for at least three months. Patients with adenocarcinoma *in situ* or invasive cancer were not eligible for observational management and were not included into this study. Initial diagnostic work-up for patients with abnormal PAP test results included the following: colposcopy, repeated PAP smears, and colposcopically guided biopsy of all suspicious lesions. Women with pathological cytology and negative or equivocal colposcopy were biopsied from all four quadrants of the uterine cervix. In selected cases endocervical curettage was performed. HPV testing using the Hybrid Capture® HPV DNA test was performed based on the physician’s decision.

Biopsy specimens were reported by the pathology service at the General Hospital of Vienna, and findings were recorded according to the CIN histologic grading system. Management and treatment decisions were the responsibility of the individual colposcopist. Follow-up examinations including clinical examination colposcopically guided biopsy, cytology+/− HPV testing and were performed every 3 to 6 months.

The primary outcome was the rate of disease regression, persistence or progression based on histologic results from colposcopically guided biopsy. Regression, progression and persistence were defined on histologic findings from colposcopically guided biopsy or conisation specimens when patients were treated with conisation at last. Regression was defined as CIN III lesion reverting to CIN II, CIN I or normal, as CIN II reverting to CIN I or normal or as CIN I reverting to normal. Complete remission was defined as any CIN lesion reverting to normal. Persistence was defined as unchanged histologic diagnosis until the study endpoint. Progression was defined as worsening from histologic diagnosis (CIN I to CIN II or CIN II to CIN III). Study endpoint was either end of observation period or time of intervention. No follow-up was performed after surgical intervention.

To account for differences within our cohort we analysed rates of remission, regression, and progression broken down by different follow-up periods, CIN grade and age in patients who were treated conservatively for at least three months.

Meta-analysis of the published data was performed according to Preferred Reporting Items for Systematic reviews and Meta-analyses for Protocols (PRISMA-P) 2015^13^. Studies were selected according to the criteria outlined below. In March 2016, a PubMed and Scopus search using the terms “cervical intraepithelial neoplasia” or “CIN” or “cervical dysplasia” and “age” and “natural history” and “regression” and “progression” was performed to identify articles on this topic published in the English literature. Only studies reporting age for regression and/or progression of histo-pathologically confirmed CIN were eligible. Studies reporting only cytological results studies including pregnant women or women with comorbidities such as cancer, infections, inflammatory diseases or autoimmune diseases were not included into the review. The authors independently screened the titles and abstracts in consideration of the inclusion criteria. We obtained full reports for all titles that appeared to meet the inclusion criteria or where there is any uncertainty. Afterwards full texts were screened and decided whether studies met the criteria. The data of our cohort were included into all analyses. Our search identified 14 articles. Of these 7 articles did not match our search criteria due to lacking histologic results. Finally 7 articles were included for quantitative meta-analysis.

Data abstracted include methodology, patient-important outcomes and demographic information. Primary outcome was the rate of progression, regression and persistence in different age groups. To ensure literature saturation, reference lists of included studies or relevant reviews identified through the search were scanned.

### Statistical analysis

Statistical analysis of data was performed using SPSS software (version 24; IBM SPSS Inc., IL, USA) and the SAS System (version 9.4; SAS Institute Inc., Cary, NC, USA). Data were described by frequencies and percentages. Pooled outcome rates and 95% confidence intervals were computed using a generalized linear model with a random effect for study. A random effects model was chosen to account for possible presence of heterogeneity, and heterogeneity was assessed by computing *I*^2^ measures as the ratio of random effect variance and total (random effect plus error) variance on the log odds scale. Rates of regression, progression, persistence and complete remission rates were compared between 5 different age groups (<25, 25 < 30, 30 < 35, 35 < 40, ≥40 years) by Cochran-Armitage linear trend tests. In a meta-regression analysis, impact of median follow-up in months and age (<30, > = 30 years) on regression rates were evaluated, again using the generalized linear model with random effect for study. Publication bias, i.e., evidence for smaller studies reporting more positive results was assessed by regressing regression, persistence and progression rates on logarithm of sample size, concluding publication bias in case of a significant (one-sided p-value <0.025) negative association for regression and persistence rates or a significant positive association for progression rates. Cochrane’s risk of bias assessment tool was applied to evaluate possible sources of bias.

Multivariable logistic regression analysis was used to estimate odds ratios (95% confidence intervals) for regression, including age as a continuous variable, and histology (CIN grade I, II, III) and HPV high-risk infection (absent, present) as adjustment factors. Missing HPV high-risk status was multiply imputed using 100 imputations. As a sensitivity analysis, complete case analysis was performed, which gave virtually identical results. A possibly nonlinear effect of age was evaluated by fitting a cubic polynomial of age, which did not significantly improve the model fit (p = 0.123 compared to linear fitting).

All data are reported as mean ± standard deviation or as relative frequency. The two-sided significance level was set to 0.05.

The datasets generated during and/or analyzed during the current study are available from the corresponding author on reasonable request.

## Electronic supplementary material

Supplementary Information
