# Supplementary material for: Influence of age on histologic outcome of cervical intraepithelial neoplasia during observational management: results from large cohort, systematic review, meta-analysis
Source: Sci Rep. 2018 Apr 23;8:6383. doi: 10.1038/s41598-018-24882-2 (PMC5913272; doi:10.1038/s41598-018-24882-2)

Influence of age on histologic outcome of cervical intraepithelial neoplasia during observational management: results from large cohort, systematic review, meta-analysis

Christine Bekos[1], Richard Schwameis[1], Georg Heinze[2], Marina Gärner[1], Christoph Grimm[1], Elmar Joura[1,4], Reinhard Horvat [3], Stephan Polterauer[1,4,*], Mariella Polterauer[1]

1. Medical University of Vienna, Comprehensive Cancer Center, Department of Obstetrics and Gynecology, Division of General Gynecology and Gynecologic Oncology
2. Center for Medical Statistics, Informatics and Intelligent Systems, Medical University of Vienna
3. Medical University of Vienna, Department of Pathology
4. Karl Landsteiner Institute for General Gynecology and Experimental Gynecologic Oncology, Austria

Corresponding author*:

Stephan Polterauer MD

Associate Professor, Department of Obstetrics and Gynecology, Medical University Vienna

Währinger Gürtel 18-20, A-1090 Vienna, Austria,

Phone: +43 1 40400 29620, FAX: +43 1 40400 29110

stephan.polterauer@meduniwien.ac.at

Supplemental material

Table S1. References of included studies, data from the current report were included into all analyses

uthor, Date	Population	N	Age, median (range)	Outcome (Regression)	Analysis	Median follow up (months)
**han, 2003 [14]**			29 (19-55)		prospective	6
	CIN II	55		58%		
	CIN III	38		47%		
**uchs, 2007 [15]**	CIN I-II	36	≤21	38.9%	retrospective	12.6
**o, 2011 [16]**			29.8		prospective	12
	CIN I	143		69.9%		
	CIN II	63		76.2%		
**ogewoning, 003 [17]**	CIN I-III	125	34.6 (19.1-54.7)	44%	randomized clinical trial	15.2
**lc Allum, 2011 8]**	CIN II	157	20.9	61.8%	retrospective	8
**loore, 2007 [19]**	CIN II	355	19.0	56.1%	retrospective	18
**loscicki, 2010 0]**	CIN II	95	20.4	68.4%	prospective	36

Table S2. Pooled analysis of studies reporting age- dependent regression rates of CIN

**Rate of regression in women <25 years**					
**Outcome parameter**	**Author**	**N**	**%**	**Lower CI**	**Upper CI**
Regression	Fuchs, 2007 [15]	36	38.9	24.800	55.100
Regression	McAllum, 2011 [18]	157	61.8	54.000	69.000
Regression	Moore, 2007 [19]	355	56.1	50.900	61.100
Regression	Moscicki, 2010 [10]	95	68.4	58.500	76.900
Regression	Bekos, 2016	141	44.7	36.700	52.900
Regression	***POOLED***	784	54.9	45.000	64.500

**Rate of regression in women <30 years**					
**Outcome parameter**	**Author**	**N**	**%**	**Lower CI**	**Upper CI**
Regression	Chan, 2003 [14]	47	44.7	31.400	58.800
Regression	Fuchs, 2007 [15]	36	38.9	24.800	55.100
Regression	Hogewoning, 2003 [17]	30	30.0	16.700	47.900
Regression	McAllum, 2011 [18]	157	61.8	54.000	69.000
Regression	Moore, 2007 [19]	355	56.1	50.900	61.100
Regression	Moscicki, 2010 [10]	95	68.4	58.500	76.900
Regression	Bekos, 2016	319	38.6	33.400	44.000
Regression	***POOLED***	1039	49.4	39.100	59.700

**Rate of regression in women <35 years**					
**Outcome parameter**	**Author**	**N**	**%**	**Lower CI**	**Upper CI**

Regression	Chan, 2003 [14]	47	44.7	31.400	58.800
Regression	Fuchs, 2007 [15]	36	38.9	24.800	55.100
Regression	Hogewoning, 2003 [17]	65	32.3	22.200	44.400
Regression	McAllum, 2011 [18]	157	61.8	54.000	69.000
Regression	Moore, 2007 [19]	355	56.1	50.900	61.100
Regression	Moscicki, 2010 [10]	95	68.4	58.500	76.900
Regression	Bekos, 2016	471	36.1	31.900	40.500
Regression	***POOLED***	1226	48.7	38.200	59.400

**Rate of regression in women ≥35 years**					
**Outcome parameter**	**Author**	**N**	**%**	**Lower CI**	**Upper CI**
Regression	Hogewoning, 2003 [17]	33	33.3	19.800	50.400
Regression	Bekos, 2016	312	26.0	21.400	31.100
Regression	***POOLED***	345	26.7	22.300	31.600

N, number; CI confidence interval;

Table S3. Pooled analysis of studies reporting age- dependent persistence rates of CIN

**Rate of persistence in women <25 years**					
**Outcome parameter**	**Author**	**N**	**%**	**Lower CI**	**Upper CI**
Persistence	Fuchs, 2007 [15]	36	8.3	2.9000	21.800
Persistence	Ho, 2011 [16]	66	42.4	31.200	54.400
Persistence	McAllum, 2011 [18]	157	38.2	31.000	46.000
Persistence	Moore, 2007 [19]	355	34.9	30.200	40.000
Persistence	Bekos, 2016	141	44.7	36.700	52.900
Persistence	***POOLED***	755	33.5	21.800	47.700

**Rate of persistence in women <30 years**					
**Outcome parameter**	**Author**	**N**	**%**	**Lower CI**	**Upper CI**
Persistence	Fuchs, 2007 [15]	36	8.3	2.9000	21.800
Persistence	Ho, 2011 [16]	66	42.4	31.200	54.400
Persistence	McAllum, 2011 [18]	157	38.2	31.000	46.000
Persistence	Moore, 2007 [19]	355	34.9	30.200	40.000
Persistence	Bekos, 2016	319	49.2	43.800	54.700
Persistence	***POOLED***	933	34.3	21.500	49.800

**Rate of persistence in women <35 years**					
**Outcome parameter**	**Author**	**N**	**%**	**Lower CI**	**Upper CI**
Persistence	Fuchs, 2007 [15]	36	8.3	2.9000	21.800
Persistence	Ho, 2011 [16]	148	36.5	29.200	44.500
Persistence	McAllum, 2011 [18]	157	38.2	31.000	46.000
Persistence	Moore, 2007 [19]	355	34.9	30.200	40.000
Persistence	Bekos, 2016	471	50.1	45.600	54.600
Persistence	***POOLED***	1167	33.5	21.200	48.500

Rate of persistence in women ≥35 years					
**Outcome parameter**	**Author**	**N**	**%**	**Lower CI**	**Upper CI**
Persistence	Ho, 2011 [16]	58	31.0	20.600	43.800
Persistence	Bekos, 2016	312	55.4	49.900	60.900
Persistence	***POOLED***	370	43.6	22.200	67.700

N, number; CI confidence interval;

Table S4. Pooled analysis of studies reporting age- dependent progression rates of CIN

Rate of progression in women <25 years					
**Outcome parameter**	**Author**	**N**	**%**	**Lower CI**	**Upper CI**
Progression	Moore, 2007 [19]	355	14.1	10.800	18.100
Progression	Moscicki, 2010 [10]	95	14.7	9.0000	23.200
Progression	Bekos, 2016	141	10.6	6.6000	16.800
Progression	***POOLED***	591	13.4	10.800	16.400

Rate of progression in women <30 years					
**Outcome parameter**	**Author**	**N**	**%**	**Lower CI**	**Upper CI**
Progression	Moore, 2007 [19]	355	14.1	10.800	18.100
Progression	Moscicki, 2010 [10]	95	14.7	9.0000	23.200
Progression	Bekos, 2016	319	12.2	9.1000	16.300
Progression	***POOLED***	769	13.4	11.200	16.000

Rate of progression in women <35 years					
**Outcome parameter**	**Author**	**N**	**%**	**Lower CI**	**Upper CI**
Progression	Moore, 2007 [19]	355	14.1	10.800	18.100
Progression	Moscicki, 2010 [10]	95	14.7	9.0000	23.200
Progression	Bekos, 2016	471	13.8	11.000	17.200
Progression	***POOLED***	921	14.0	11.900	16.400

Rate of progression in women ≥35 years					
**Outcome parameter**	**Author**	**N**	**%**	**Lower CI**	**Upper CI**
Progression	Bekos, 2016	312	18.6	14.700	23.300
Progression	***POOLED***	312	18.6	14.600	23.300

N, number; CI confidence interval;

Table S5. Cochrane risk of bias assessment tool for study Moscicki AB et al., 2010;

Domain	Support for judgement	Review authors' judgement
Selection bias		
Random sequence generation	does not apply	does not apply
Allocation concealment	does not apply	does not apply

Performance bias		
Blinding of participants and personnel	Blinding of participants: not possible as participants are not blind for their age.<br>Blinding of personnel: personnel cannot be blind to age of participants.	Low risk
Detection bias		
Blinding of outcome assessment	Histological specimens were reviewed independently by 2 (CIN2) or 3 (CIN3) separate pathologist. It is unclear whether pathologists were blinded to age of patients.	Low risk
Attrition bias		
Incomplete outcome data	Heterogenous outcome data because of mean (SD) length of follow up of 27.4 (SD 11.6) with a range of 3.8 to 46.8 months. 68% of patients had an exit biopsy. The remaining patients either refused or did not come for the final visit.	*High risk*
Reporting bias		
Selective reporting	Reported outcome was due to histological diagnosis of cervical biopsy. Progression and regression are reported.	*Low risk*
Other bias		
Other sources of bias	Inclusion criteria: 13-24 years, cervical cytology showing ASCUS, LSIL or HSIL, CIN 2;<br><br>Exclusion criteria: previous treatment of CIN, immunosuppression, pregnancy	*Low risk*

Table S6. Cochrane risk of bias assessment tool for study Ho G et al., 2011;

Domain	Support for judgement	Review authors' judgement
Selection bias		
Random sequence generation	does not apply	does not apply
Allocation concealment	does not apply	does not apply

Performance bias		
Blinding of participants and personnel	Blinding of participants: not possible as participants are not blind for their age.<br>Blinding of personnel: pathologists were blinded to age of participants.	Low risk
Detection bias		
Blinding of outcome assessment	Histological specimens were reviewed independently by 2 separate pathologists, who were blinded to the paired biopsies.	Low risk
Attrition bias		
Incomplete outcome data	Homogenous outcome data: 12 month follow up.	*Low risk*
Reporting bias		
Selective reporting	Reported outcome was due to histological diagnosis of cervical biopsy. Regression, persistence and progression are reported.	*Low risk*
Other bias		
Other sources of bias	Inclusion criteria: age >18 years, CIN 1 and 2, no prior CIN diagnosis or treatment, satisfactory colposcopy, not pregnant, recruitment 4 months after CIN diagnosis	*Low risk*

Table S7. Cochrane risk of bias assessment tool for study Chan JK et al., 2003;

Domain	Support for judgement	Review authors' judgement
Selection bias		
Random sequence generation	does not apply	does not apply
Allocation concealment	does not apply	does not apply
Performance bias		
Blinding of participants and personnel	Blinding of participants: not possible as participants are not blind for their age.<br>Blinding of personnel: personnel cannot be blind to age of participants.	High risk
Detection bias		
Blinding of outcome assessment	Histological specimens were reviewed	Low risk

	independently by 2 separate pathologists. It is unclear whether pathologists were blinded to age of patients.	
Attrition bias		
Incomplete outcome data	Homogenous outcome data: 6 month follow up.	*High risk*
Reporting bias		
Selective reporting	Reported outcome was due to histological diagnosis of cervical biopsy. Only Regression is reported. All patients with persistent CIN 3 or Progression to CIN3 were excluded.	*High risk*
Other bias		
Other sources of bias	Inclusion criteria: age >17 years, CIN 2 and 3, negative endocervical curettage;	*Low risk*

Table S8. Cochrane risk of bias assessment tool for study McAllum B et al., 2011;

Domain	Support for judgement	Review authors' judgement
Selection bias		
Random sequence generation	does not apply	does not apply
Allocation concealment	does not apply	does not apply
Performance bias		
Blinding of participants and personnel	Blinding of participants: not possible as participants are not blind for their age.<br>Blinding of personnel: personnel cannot be blind to age of participants.	High risk
Detection bias		
Blinding of outcome assessment	Histological specimens were reported by pathologic services at each of the 3 participating colposcopy clinics.	High risk
Attrition bias		
Incomplete outcome data	Heterogenous outcome data were reported at 4-9, 10-15, 16-20 and 21-24month intervals.	*High risk*
Reporting bias		
Selective reporting	Reported outcome was due to histological diagnosis of cervical biopsy. Regression,	*Low risk*

	persistence and progression are reported.	
Other bias		
Other sources of bias	Inclusion criteria: age <25 years, CIN 2;	*Low risk*

Table S9. Cochrane risk of bias assessment tool for study Hogewoning C et al., 2003;

Domain	Support for judgement	Review authors' judgement
Selection bias		
Random sequence generation	does not apply	does not apply
Allocation concealment	does not apply	does not apply
Performance bias		
Blinding of participants and personnel	Blinding of participants: not possible as participants are not blind for their age.<br>Blinding of personnel: colposcopic findings were documented by photographs. These photographs were reviewed by an experienced colposcopist blinded of any clinical data. In case of discrepancy a consensus diagnosis was made.	Low risk
Detection bias		
Blinding of outcome assessment	Colposcopic findings were documented by photographs. These photographs were reviewed by an experienced colposcopist blinded of any clinical data. In case of discrepancy a consensus diagnosis was made.	Low risk
Attrition bias		
Incomplete outcome data	Heterogenous outcome data: median follow up time of 15.2 (range 3.0-85.4)	*High risk*
Reporting bias		
Selective reporting	Reported outcome was due to histological diagnosis of cervical biopsy. Regression, persistence and progression	*Low risk*

	are reported.	
Other bias		
Other sources of bias	Inclusion criteria: CIN 1-3, no prior treatment	*Low risk*

Table S10. Cochrane risk of bias assessment tool for study Fuchs K et al., 2007;

Domain	Support for judgement	Review authors' judgement
Selection bias		
Random sequence generation	does not apply	does not apply
Allocation concealment	does not apply	does not apply
Performance bias		
Blinding of participants and personnel	Blinding of participants: not possible as participants are not blind for their age.<br>Blinding of personnel: personnel cannot be blind to age of participants.	High risk
Detection bias		
Blinding of outcome assessment	Histologic specimens were evaluated by the local hospital's pathologists. No information about blinded specimens.	High risk
Attrition bias		
Incomplete outcome data	Outcome data: follow up time of 4-6 months	*High risk*
Reporting bias		
Selective reporting	Reported outcome was due to histological diagnosis of cervical biopsy. Regression, persistence and progression are reported.	*Low risk*
Other bias		
Other sources of bias	Inclusion criteria: age <21 years, CIN 1 and 2	*Low risk*

Table S11. Cochrane risk of bias assessment tool for study Moore K al., 2007;

Domain	Support for judgement	Review authors' judgement
Selection bias		
Random sequence generation	does not apply	does not apply
Allocation concealment	does not apply	does not apply
Performance bias		
Blinding of participants and personnel	Blinding of participants: not possible as	Low risk

	participants are not blind for their age.<br>Blinding of personnel: personnel cannot be blind to age of participants.	
Detection bias		
Blinding of outcome assessment	No information about blinded outcome assessment.	High risk
Attrition bias		
Incomplete outcome data	Outcome data: median follow up time of 18 months, patients were seen every 4-6 months	*Low risk*
Reporting bias		
Selective reporting	Reported outcome was due to histological diagnosis of cervical biopsy. Regression, persistence and progression are reported.	*Low risk*
Other bias		
Other sources of bias	Inclusion criteria: age <21 years, CIN 1 and 2	*Low risk*

Table S12. Risk of bias summary table

Study	Attrition bias (high if length of follow-up <12 months, low if ≥12 months, ? if no statement on length of follow-up found)	Selective reporting (high/low/?)	Detection bias (high/low/?)
**Moscicki AB et al., 2010**	High risk	Low risk	Low risk
**Ho G et al., 2011**	Low risk	Low risk	Low risk
**Chan JK et al., 2003**	High risk	High risk	Low Risk
**McAllum B et al., 2011**	High risk	Low risk	High Risk
**Hogewoning C et al., 2003**	High risk	Low Risk	Low Risk
**Fuchs K et al., 2007**	High risk	Low risk	High risk
**Moore K al., 2007**	Low risk	Low risk	High risk

Figure S1. A positive correlation was found between sample size and regression rates, indicating the absence of presence of publication bias (regression coefficient +7.7% per doubling of sample size, one-sided p=0.997).

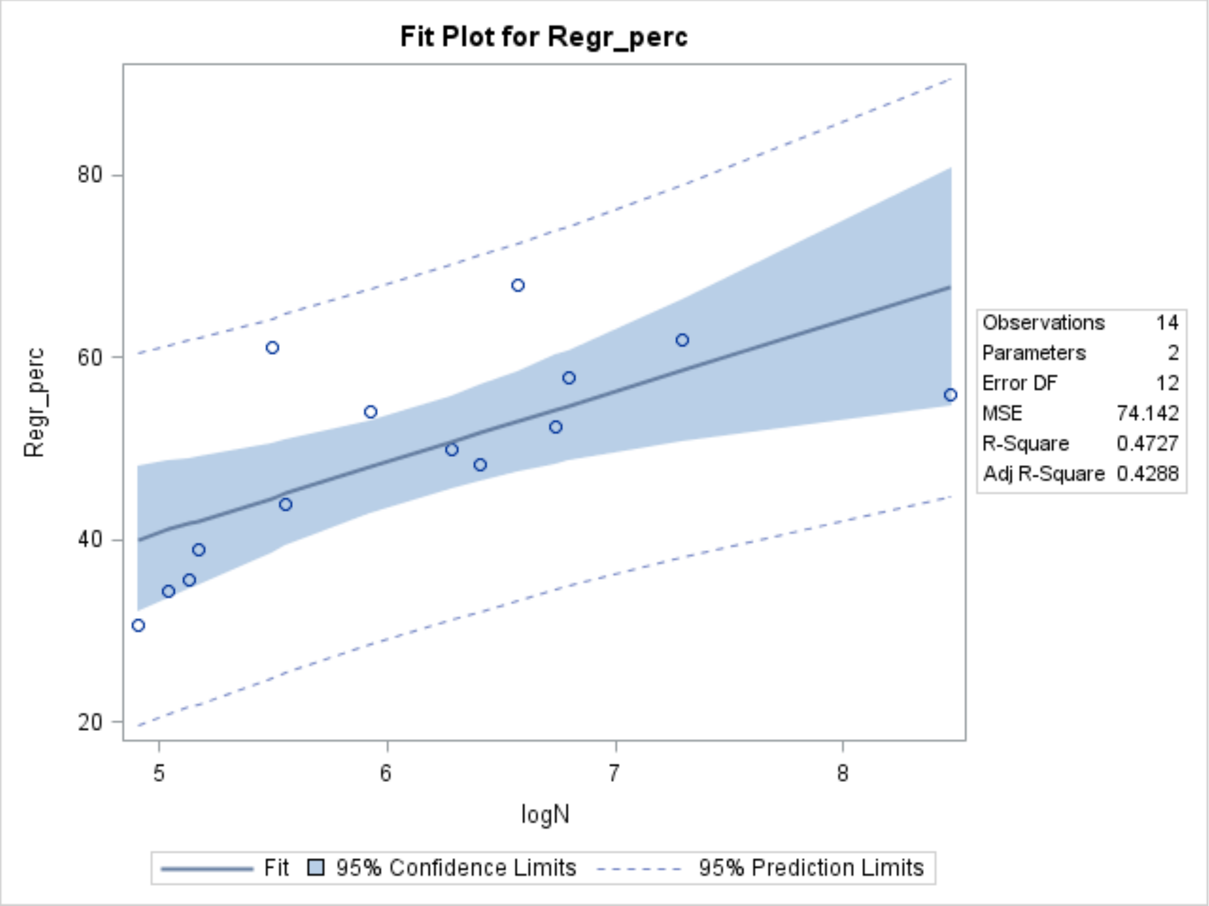

Figure S2. Persistence increased by 4.6% per doubling of sample size (P=0.906).

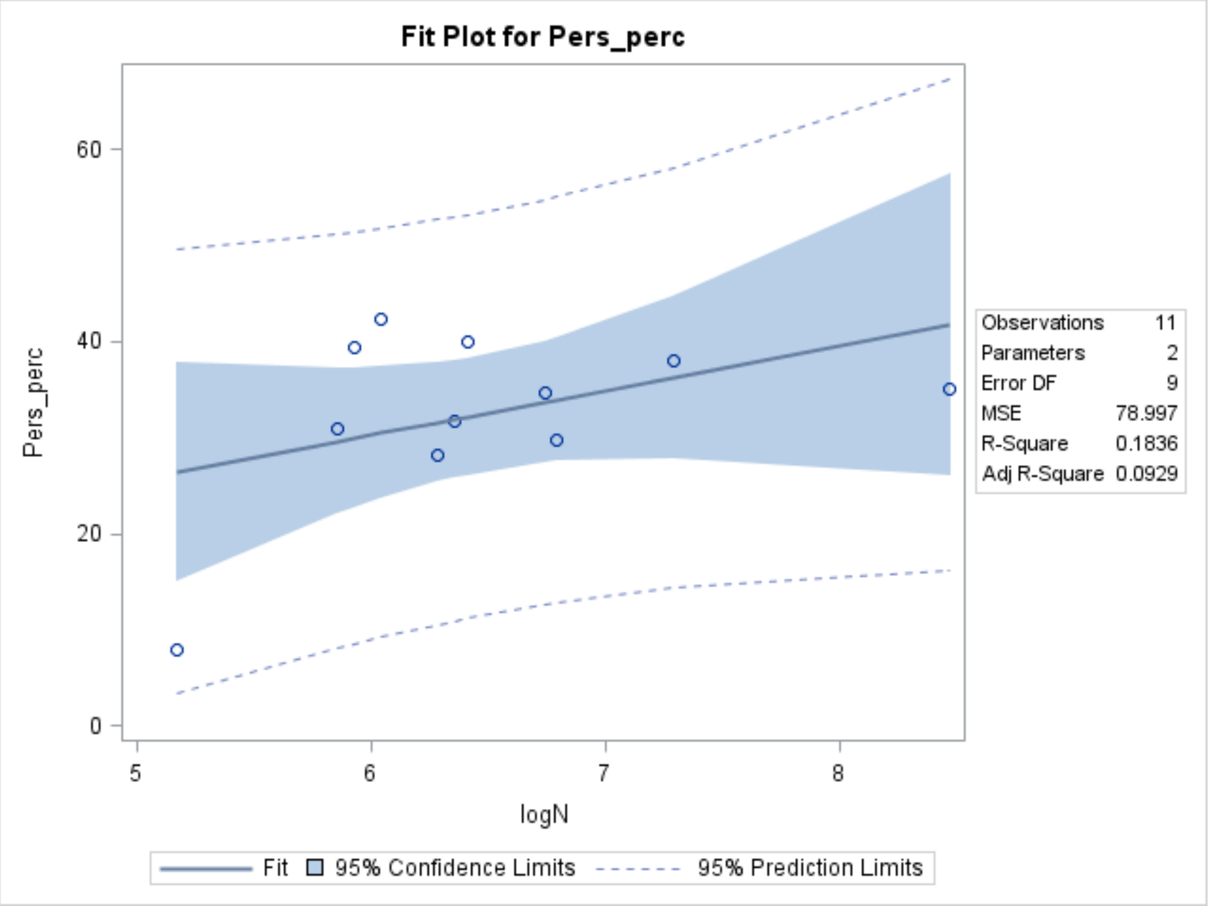

Figure S3. Progression rates were not significantly associated with sample size (+0.73% per doubling of sample size, p=0.389).

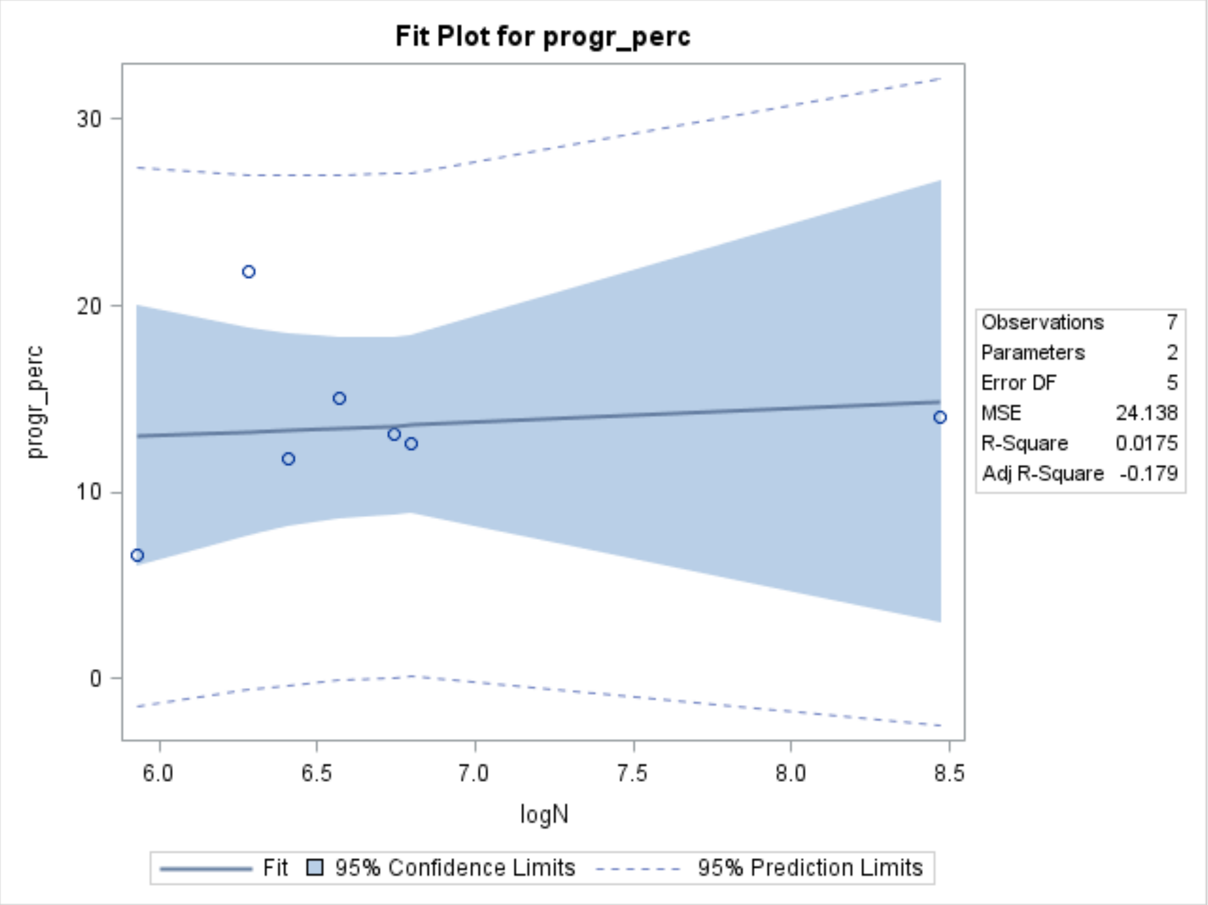

Supplement: Supplementary file 1 — Supplementary Information [file 41598_2018_24882_MOESM1_ESM.pdf]
